# Learning curve associated with thoraco-laparoscopic esophagectomy for esophageal cancer patients in the prone position

**DOI:** 10.1186/s13019-020-01161-8

**Published:** 2020-05-27

**Authors:** Tao Wang, Mu-yuan Ma, Bo Wu, Yang Zhao, Xiao-feng Ye, Tao Li

**Affiliations:** Department of Sugicial Oncology II, General Hospital of Ningxia Medical University, Yinchuan, 750004 Ningxia China

**Keywords:** Esophagectomy of esophageal cancer, Prone position, Learning curve

## Abstract

**Objective:**

To observe the surgical index at the different learning stages of thoraco-laparoscopic esophagectomy in the prone position for esophageal cancer and to investigate the learning curve of this surgical procedure.

**Methods:**

Sixty thoraco-laparoscopic esophagectomies in the prone position for esophageal cancer conducted by the same group of surgeons between January 2014 and December 2015 were retrospectively analyzed. The surgeries were divided into 5 groups, A, B, C, D, and E, in chronological order. The duration of surgery, intraoperative blood loss, total number of lymph nodes removed, rate of the intraoperative conversion to open surgery, complication rate, and length of postoperative hospitalization were recorded and analyzed.

**Results:**

The general information of the patients did not significantly differ among the 5 groups (*P* > 0.05). The duration of surgery, intraoperative blood loss, number of lymph node removed, rate of intraoperative conversion to open surgery, and number of injuries to the recurrent laryngeal nerve all significantly differed (*P* < 0.05). The rates of postoperative pulmonary infection, anastomotic fistula, pneumothorax, and hospitalization did not significantly differ (*P* > 0.05).

**Conclusion:**

Thoracic physicians with some endoscopic experience can meet the requirements of the thoraco-laparoscopic esophagectomy in the prone position for esophageal cancer after completing 24–30 surgeries.

## Introduction

Esophagectomy is the main treatment for esophageal cancer [1, 2], but traditional open surgery is associated with significant trauma and various complications that affect the efficacy of treatment. In the 1990s, Pellegrini [3] reported the completion of video-assisted thoracoscopic esophagectomy for esophageal cancer. Because video-assisted thoracoscopic esophagectomy avoids long incisions in the chest and abdomen and cutting of the ribs, it causes minimal intraoperative trauma, blood loss, postoperative pain, and complications. Therefore, this approach has been widely accepted [4, 5].

Based on initial thoracoscopic assistance, video-assisted thoracoscopic esophagectomy has been gradually developed into a combined thoraco-laparoscopic esophagectomy technique. Because a prone position provides good surgical field exposure and allows for complete lymph node dissection, thoraco-laparoscopic esophagectomies have been carried out in this position [6, 7]. A thoraco-laparoscopic esophagectomy for the treatment of esophageal cancer requires the surgeon to master both thoracoscopy and laparoscopy techniques; therefore, the surgery is more difficult, and its risk is relatively high. To completely master this surgical procedure and accumulate enough experience, surgeons undergo must go a learning curve of exploration, consolidation, improvement, and perfection [8]. The present study retrospectively analyzes 60 thoraco-laparoscopic esophagectomies in the prone position for esophageal cancer conducted by the same team of surgeons and analyzes the surgical indices during different stages to investigate the learning curve associated with this surgical procedure.

## Patients and methods

### General information of patients

Sixty patients who underwent a thoraco-laparoscopic esophagectomy in the prone position for esophageal cancer conducted by the same team of surgeons between January 2014 and December 2015 were selected. Of these patients, 48 were males and 12 were females, and their average age was 58.6 ± 4.6 years. Six patients were diagnosed with upper thoracic cancer, 39 patients were diagnosed with middle thoracic cancer, and 15 patients were diagnosed with lower thoracic cancer. Furthermore, 19 patients were diagnosed with stage I disease, 26 patients were diagnosed with stage II disease, 15 patients were diagnosed with stage III disease, and none of the patients had stage IV disease. Eleven patients presented with preoperative hypertension, 10 had diabetes, and 28 had a history of smoking. The postoperative endoscopic pathological diagnosis of all patients was esophageal squamous cell carcinoma. Preoperative relevant examinations indicated that none of the patients had an obvious surgical contraindication and that the tumor did not exhibit significant external invasion or distant metastases. All patients signed surgical consent before the operation.

### Surgical team

All the surgery was completed with professor Tao Li. The surgeon has the experience for independently completed 50 cases of open esophageal cancer radical operation and mastered the laparoscopic radical gastrectomy. Doctor Tao Wang was fixed as the first assistant. Doctor Mu-yuan Ma was fixed as the second assistant.

### Surgical procedures

The patients were administered general anesthesia and underwent double-lumen endotracheal intubation. The patient was then fixed in a prone position. The patient’s right side was elevated 30 degrees, the right upper limb was in full abduction, and the patient then underwent routine disinfection and draping.

#### Thoracic surgery

A 1 cm incision was made at the anterior axillary line in the 7th intercostal space, and a 12 mm trocar thoracoscope was inserted to observe possible pleural adhesions. In cases of significant adhesion that could not be separated, the patient was immediately converted to a thoracotomy. Next, CO_2_ was filled to a pressure of 8 cm Hg to establish artificial pneumothorax, and the right lung was completely collapses. In addition, a 5 mm trocar was inserted at the medial subscapularis, a 12 mm trocar was inserted at the posterior axillary line in the 9th intercostal space, and a 5 mm trocar was inserted at the posterior axillary line in the 3rd intercostal space, which was used as the surgical port. The mediastinal pleura was opened to observe whether the tumor had invaded or adhered to the surrounding blood vessels, trachea, and pericardium. In case of external invasion or metastasis, the patient was immediately converted to a thoracotomy or the surgery was terminated. The azygos vein was stripped, clipped, and cut; the thoracic esophagus was cut with an ultrasonic knife, and the small arteries surrounding the esophagus were individually cut; moving upwards to reach the top of the right chest and going downwards to reach the diaphragmatic aperture, the lymph nodes next to the left and right recurrent laryngeal nerves, esophagus, and under the carina were removed while taking care to protect the thoracic duct. Bleeding was stopped, and after ensuring the absence of active bleeding points in the thoracic cavity and no air leakage on the surface of the lungs, trachea, and bronchus, the lungs were expanded. A thoracic duct, which was connected to a water-sealed drainage bottle, was kept at the posterior axillary line in the 9th intercostal space, and the various operation ports were sutured.

#### Abdominal surgery

The patient was moved to a supine, reverse Trendelenburg position, the shoulders were elevated with padding, and the head was turned to the right side. A 1 cm incision was made below the umbilicus to insert an insufflation needle, and the cavity was filled with CO_2_ to a pressure of 14 cm Hg to establish an artificial pneumoperitoneum. A 12 mm trocar was inserted from the incision beneath the umbilicus as the observation port for the laparoscope, and the laparoscope was used to look for abdominal organ adhesions, ascites, and masses in the liver and spleen. A 5 mm trocar was inserted on the cross point of the costal arch and anterior axillary line on both sides, and a 12 mm trocar was placed at the intersection of the right clavicle midline and 3 cm above the umbilicus. The hepatogastric ligament was cut under the liver, and the liver was separated along the small curvature of the stomach to the right side of the abdominal esophagus. The left gastric artery was separated and cut off with an ultrasonic knife. An ultrasonic knife was also used to cut the gastrocolic ligament along the greater curvature of the stomach, cut and ligate the left gastroepiploic artery and vein, and individually cut the short gastric vessels. The greater curvature of the stomach to the left of the left side of the abdominal esophagus was fully separated, and the omentum and vascular arch were preserved with the greater curvature side. The lymph nodes next to the left gastric artery, the lesser curvature lymph nodes, and the lymph nodes next to the common hepatic artery were cleaned. The endoscope and the trocar were removed, and a 5 cm median incision was made below the xiphoid in the upper abdomen. An incision was made in the abdomen, and the stomach was removed. The esophagus was separated at the junction of esophagus and stomach, and the stump of the esophagus was ligated for protection; the stomach was pulled out from the abdominal incision, and a disposable cutter-stapler was used to cut off the lesser curvature of the stomach parallel to the greater curvature of the stomach from the fundus to the incisura angularis to shape the stomach into a tub; the stump was then closed with an interrupted suture with thread and embedding.

#### Neck surgery

An incision was made through the front edge of the left sternocleidomastoid muscle, and the scapulae muscle and the middle thyroid vein were cut to expose the esophagus. Blunt and sharp dissections of the esophagus were alternated, and care was taken to protect the recurrent laryngeal nerve while moving downwards to the top of right chest; the esophagus was pulled out through the esophageal bed and left neck incision, and dissection was continued the middle of the thyroid; an incision was made in the cervical esophagus, and a disposable needle was inserted pointing to the seat of a curved anastomosis stapler; ligation was performed with a purse-string suture; the prepared tubular stomach was pulled out to the neck through the esophageal bed and left neck incision, and a disposable curved anastomosis stapler was used to perform a mechanical anastomosis of the tubular stomach-left neck end of esophagus. After ensuring that both cut rings are integral, No.1 thread was used for an interrupted suture of the seromuscular layer to embed the anastomosis. The stomach tube was placed into the predetermined location, and a disposable cutter-stapler was used to close the opening at the fundus of the tubular stomach. A No.1 interrupted suture of the seromuscular layer was used to embed the opening at the fundus of the stomach. Diluted iodophor solution was used to rinse the neck, and a negative-pressure drainage tube was maintained while suturing the incision at the neck. The abdominal cavity was carefully examined, and bleeding was stopped. After comparing the gauze and number of instruments to the inventory, the abdomen was closed layer by layer.

All patients underwent a mechanical anastomosis of the esophagus, and a tubular stomach was generated at the neck. The surgeons have a wealth of experience in open surgery for esophageal cancer and some endoscopic surgical skill. In the early stages, the surgery was performed under the guidance of a physician with endoscopic experience.

The 60 patients were evenly and chronologically divided into 5 groups (A, B, C, D, and E) of 12 patients each. Information on surgical duration, intraoperative blood loss, total number of removed lymph nodes, rate of intraoperative conversion to open surgery, complication rate (including pulmonary infection, anastomotic fistula, recurrent laryngeal nerve injury, and postoperative pneumothorax), and length of postoperative hospitalization was chronologically collected.

## Results

The general clinical information, including age, gender, tumor position, tumor stage, preoperative complications (hypertension or diabetes), and smoking history, did not significantly differ between the 5 groups of patients.

### Comparison of surgical results

The average duration of surgery significantly differed between the 5 groups (*P* < 0.05); specifically, the surgical duration was significantly longer in groups A and B than in the other 3 groups (C, D, and E) (*P* > 0.05), which did not significantly differ in surgical duration (P > 0.05, see Table 1). Moreover, the average blood loss significantly differed between groups (*P* = 0. 001); specifically, it did not significantly differ between groups A and B (*P* = 0.952), but these two groups significantly differed from groups C, D and E (*P* < 0.05). Blood loss did not significantly differ among groups C, D and E (*P* > 0.05, see Table 2). Intraoperative conversion to open surgery was significantly more common in groups A and B than in groups C, D, and E (*P* < 0.05). Moreover, the number of removed lymph nodes significantly differed among the 5 groups (*P* > 0.05, see Table 2). Specifically, significantly fewer lymph nodes were removed in groups A and B than in groups C, D, and E (*P* < 0.05), whereas the number of removed lymph nodes did not significantly differ among groups C, D, and E (*P* > 0.05, see Table 3). The average postoperative hospitalization time did not significantly differ among the 5 groups (*P* = 0.0738). In terms of postoperative complications in the 5 groups, 24 patients developed pulmonary infection, 5 patients developed anastomotic leakage, 1 patient developed a tubular stomach stump fistula, and 7 patients developed postoperative pneumothorax; these rates did not significantly differ between groups (*P* > 0.05). A total of 4 patients developed recurrent laryngeal nerve injury, and this incidence significantly differed between groups (*P* < 0.05).

**Table 1:** General information of patients

	A	B	C	D	E	*P*
gender (male/female)	9/3	8/4	10/2	10/2	11/1	> 0.05
Age (years)	61.2 ± 5.4	57.3 ± 4.3	58.6 ± 4.6	57.5 ± 6.4	60.8 ± 3.7	> 0.05
Tumor location						> 0.05
upper	1	0	2	2	1	
middle	8	9	7	8	7	
lower	3	3	3	2	4	
Clinical stages						> 0.05
I	3	4	5	4	3	
II	6	5	4	6	5	
III	3	3	3	2	4	
IV	0	0	0	0	0	
hypertension	3	1	3	2	2	> 0.05
diabetes	2	3	1	2	2	> 0.05
Smoking history	5	4	6	6	7	> 0.05

**Table 2:** Comparison of surgical indices of each group

	A	B	C	D	E	*P*
Surgery duration (min)	504.5 ± 8.5	499 ± 9.5	384 ± 40.5	341.7 ± 24.6	330.8 ± 15.4	< 0.05
Intraoperative blood loss (ml)	351.3 ± 29.6	325 ± 27.1	254.2 ± 25.0	204.2 ± 19.3	168.3 ± 19.0	< 0.05
Number of removed lymph nodes	8.5 ± 2	8.6 ± 1.6	10.9 ± 1.6	12.8 ± 1.9	14.3 ± 2.5	< 0.05
Intraoperative conversion to open surgery	3	2	0	1	0	< 0.05
Postoperative hospitalization time (d)	14.7 ± 3.5	13.9 ± 4.4	12.3 ± 5.4	12.3 + 4.0	10.8 ± 1.4	> 0.05

**Table 3:** Comparison of postoperative complications in each group

Postoperative complications	A	B	C	D	E	*P*
Pulmonary infection	7	6	4	4	3	> 0.05
anastomotic leakage	2	1	1	2	0	> 0.05
recurrent laryngeal nerve injury	1	2	0	1	0	< 0.05
postoperative pneumothorax	2	3	1	2	1	> 0.05

## Discussion

### The advantage of thoraco-laparoscopic esophagectomy in the prone position for esophageal cancer

Luketich et al. [9] were the first to report the combined thoraco-laparoscopic esophagectomy technique, which provided a new strategy for the minimally invasive treatment of esophageal cancer. Thoraco-laparoscopic esophagectomy has several advantages, such as minimal surgical trauma, less intraoperative blood loss than traditional approaches, and a short hospitalization time, and it allows a thorough cleaning of lymph nodes [10–12]. Moreover, the efficacy of this surgical procedure has been proven to be equivalent or better than that of traditional surgery [13, 14]. In 2006, Palanivelu et al. [15] reported the encouraging results of a study of thoracoscopic esophagectomy in the prone position for 130 patients with esophageal cancer, which attracted the attention of esophageal physicians [11, 16]. This surgical procedure has the following characteristics. 1. The right lung droops naturally due to the gravity and does not require traction, and the surgical field has a good exposure; 2. in the prone position, lymph nodes under the diaphragmatic aperture, lateral posterior mediastinum, thoracic inlet, esophageal hiatus, inferior pulmonary ligament, and carina are easily cleaned; 3. under the action of gravity, the leaked blood accumulates below the thoracic cavity, which reduces the intraoperative use of an aspirator, ensures the continuity of the surgery, and shortens the operation time; and 4. lastly, the surgical port is at the level of the surgeon’s elbow, and the application of long endoscopic instruments for the separation has ergonomic advantages [17].

### Determination of the learning curve of the thoraco-laparoscopic esophagectomy in the prone position for esophageal cancer

Thoraco-laparoscopic esophagectomy in the prone position for esophageal cancer requires the surgeon to conduct both a thoracoscopy and a laparoscopy, placing strict requirements on the surgeon’s skill. In particular, operating a laparoscope is very difficult for a thoracic surgeon. In addition, because the surgical time is long during the initial learning stage, the advantages of thoraco-laparoscopic esophagectomy are not easily revealed: the treatment is not minimally invasive, and the complications do not decrease and may even increase compared with open surgery. Therefore, widely applying this technique is difficult, and surgeons must undergo a long process of learning, exploring, and mastering to gain sufficient skills to eventually achieve satisfactory results.

The long learning process will be associated with corresponding changes in surgical duration, intraoperative blood loss, rate of intraoperative conversion to open surgery, postoperative complications, and postoperative hospitalization time [18]. Accordingly, the proficiency of the surgeon was observed to evaluate the ability of the surgeon to successfully finish the surgery, ensure the quality of the surgery, and provide the patient with the best treatment possible [19]. Normally, the number of surgeries required during this stage is used to assess the learning curve. For example, some studies have shown that approximately 30 thoracoscopic esophageal surgeries are needed for the physicians to consistently perform the surgery well [20].

The present study summarized and analyzed the results of 60 thoraco-laparoscopic esophagectomies in the prone position for esophageal cancer that were consecutively performed by the same treatment group. The physicians had a wealth of experience in open surgery for esophageal cancer but had never completed a combined thoraco-laparoscopic esophagectomy for esophageal cancer. During the early stage of the learning curve, the intraoperative operation time, blood loss, number of cleaned lymph nodes, and other indices of groups A and B were worse than the corresponding indices of groups C, D, and E, indicating that the surgeons in the early surgery groups lacked experience in case selection and teamwork. Moreover, they were not familiar with the anatomical relationship under the endoscope, and they were not proficient in using the endoscopic instruments, which resulted in a slow surgery that was prone to result in bleeding and few cleaned lymph nodes. Even excessive bleeding and other emergencies occurred during surgery, which could not be resolved under the endoscope; eventually 5 patients were converted to open surgery. Patients in groups A and B remained in the hospital for a few days after the surgery, and the rates of postoperative pulmonary infection, anastomotic fistula, and pneumothorax did not significantly differ between groups A and B and groups C, D and E. This lack of difference was due to the wealth of experience of the surgeons in open surgery, which allowed them to effectively prevent common postoperative complications and ensure good surgical outcomes. Moreover, the incidence of recurrent laryngeal nerve injury was higher in groups A and B than in groups C, D, and E, possibly because the physicians in the early stage were not familiar with the thoracic anatomy of the bilateral recurrent laryngeal nerve. Thus, they may have accidentally injured the nerve when cleaning the lymph nodes. Subsequently, as the surgeons gained experience, the number of nerve injuries decreased [21]. However, the total incidence of recurrent laryngeal nerve injury was 6.7%, which is consistent with previous reports. The indices groups C, D, and E were significantly better than those of the first 2 groups, suggesting that the surgical experience and skill of the medical group had reached a relatively stable state that met the requirements of the use of a combined thoraco-laparoscopic esophagectomy for the treatment of esophageal cancer. Based on the above data, we conclude that the learning curve associated with combined thoraco-laparoscopic esophagectomy for esophageal cancer consists of approximately 24–30 surgeries.

### The factors influencing the learning curve

The physicians should improve surgical techniques as soon as possible to shorten the learning process, which is very important to allow the patients to benefit as early as possible. The following aspects should be noted during the learning process. 1. Extensive experience in open esophageal surgery is necessary for successful combined thoraco-laparoscopic esophagectomy for esophageal cancer. The physicians in this group have completed several hundred open surgeries for esophageal cancer; therefore, when they encountered complications during endoscopic surgery, they were immediately able to perform an open surgery to ensure the successful completion of a surgery. In the early stage, standardized endoscopic training and surgical observation is the basis for the development of endoscopic surgery. Before carrying out this surgery, our team had spent half a year observing and training in esophageal cancer endoscopic surgery and had a more comprehensive understanding of the procedure, skills, and complicated protocols of this surgery, which ensured the smooth development of the new surgery. Our department had previously developed a laparoscopic resection technique for gastric cancer, which helped to employ the combined thoraco-laparoscopic esophagectomy. 2. Excellent and reliable instruments for the combined thoraco-laparoscopic esophagectomy for the treatment of esophageal cancer are very important, and the physicians should be able to reasonably and flexibly use the instruments to avoid causing accidental injury and increasing the occurrence of postoperative complications. Moreover, a fixed team and tacit understanding between members can improve efficiency, ensure surgical safety, and shorten the learning curve. In particular, the endoscope operator is very important because a good exposure of the surgical field is required for a successful surgery. In addition, the physicians should also have experience in operating the endoscope to correctly direct the assistants in the operation. 3. The combined thoraco-laparoscopy for esophageal surgery takes a long time and is associated with high risk; thus, the surgical team must be prepared to address these risks. Prior to the surgery, the patient’s condition must be carefully analyzed, and surgical indications must be strictly followed. For patients suffering from late-stage disease or contraindications, traditional open surgery, with which the physicians are familiar, should be selected. In cases of serious adverse events during an endoscopic surgery, the surgery must be promptly converted to open surgery. 4. The anatomy must be continuously studied under an endoscope, and the surgery procedure must be optimized to reduce and eliminate unnecessary action, avoid complications, and further shorten the surgical time. After the surgery, the team should carefully summarize the experience in an effort to standardize, program, and simplify the thoraco-laparoscopy surgical process and establish a relatively fixed surgical model and operation procedure for the team. Using continuous summarization and improvement, the physicians can rapidly and steadily advance through this learning curve.

## Conclusion

The present study finds that a thoracic surgeon needs to complete approximately 24–30 thoraco-laparoscopic esophagectomies in the prone position for the treatment of esophageal cancer to fully meet the requirements of this type of surgery. However, the present study is limited in that it only describes the learning curve associated with this technique based on surgeon’s experience; experiences differ among physicians, and the learning curve may consequently differ by surgeon. The present study only aims to provide a reference and an experience summary for the development of this type of surgery.

## Data Availability

The datasets used and analyzed during the current study are available from the corresponding author on reasonable request.
